# Growth and characterization of textured well-faceted ZnO on planar Si(100), planar Si(111), and textured Si(100) substrates for solar cell applications

**DOI:** 10.3762/bjnano.8.194

**Published:** 2017-09-15

**Authors:** Chin-Yi Tsai, Jyong-Di Lai, Shih-Wei Feng, Chien-Jung Huang, Chien-Hsun Chen, Fann-Wei Yang, Hsiang-Chen Wang, Li-Wei Tu

**Affiliations:** 1 Department of Applied Physics, National University of Kaohsiung, No.700, Kaohsiung University Road, Nanzih Dist., Kaohsiung 811, Taiwan, R.O.C; 2 Green Energy and Environment Research Labs, Industrial Technology Research Institute, Hsinchu, Taiwan, R.O.C; 3 Department of Electronic Engineering, Southern Taiwan University of Science and Technology, Tainan, Taiwan, R.O.C; 4 Graduate Institute of Opto-Mechatronics, National Chung Cheng University, Chia-yi, Taiwan, R.O.C; 5 Department of Physics and Center for Nanoscience and Nanotechnology, National Sun Yat-Sen University, Kaohsiung, Taiwan, R.O.C

**Keywords:** atomic force microscopy, cathode luminescence, scanning electron microscopy, silicon solar cells, transparent conducting oxide, X-ray diffraction, ZnO

## Abstract

In this work, textured, well-faceted ZnO materials grown on planar Si(100), planar Si(111), and textured Si(100) substrates by low-pressure chemical vapor deposition (LPCVD) were analyzed by X-ray diffraction (XRD), scanning electron microscopy (SEM), atomic force microscopy (AFM), and cathode luminescence (CL) measurements. The results show that ZnO grown on planar Si(100), planar Si(111), and textured Si(100) substrates favor the growth of ZnO(110) ridge-like, ZnO(002) pyramid-like, and ZnO(101) pyramidal-tip structures, respectively. This could be attributed to the constraints of the lattice mismatch between the ZnO and Si unit cells. The average grain size of ZnO on the planar Si(100) substrate is slightly larger than that on the planar Si(111) substrate, while both of them are much larger than that on the textured Si(100) substrate. The average grain sizes (about 10–50 nm) of the ZnO grown on the different silicon substrates decreases with the increase of their strains. These results are shown to strongly correlate with the results from the SEM, AFM, and CL as well. The reflectance spectra of these three samples show that the antireflection function provided by theses samples mostly results from the nanometer-scaled texture of the ZnO films, while the micrometer-scaled texture of the Si substrate has a limited contribution. The results of this work provide important information for optimized growth of textured and well-faceted ZnO grown on wafer-based silicon solar cells and can be utilized for efficiency enhancement and optimization of device materials and structures, such as heterojunction with intrinsic thin layer (HIT) solar cells.

## Introduction

Transparent conductive oxides (TCOs), with both high electrical conductivity and optical transparency, could be used as a replacement for the metal contact in semiconductor devices. When applied to solar cells, it can eliminate the optical shading effect induced by the conventional metal contact thereby effectively increasing solar cell photocurrent and efficiency. Granular ZnO thin films grown by low pressure chemical vapor deposition (LPCVD) can act as good TCOs for thin film silicon solar cells [1–10]. This is mainly due to its high transparency over the visible and near-infrared (NIR) wavelength range, lower electrical resistivity (down to 1 × 10^−3^ Ω·cm), and the light-trapping capability due to its granular structure [3]. Similarly, ZnO grown on bulk silicon substrates and their application to wafer-based silicon solar cells is an interesting issue and an important subject [3]. In such a case, these materials potentially not only act as metal contacts to eliminate the blocking of the incident sunlight from the metals, but they can also serve the function of antireflection coating (ARC) films, given proper design of the film thickness. A ZnO thin film with appropriate doping could potentially act as the emitter with a Si substrate base to form a heterostructure solar cell. Therefore, in the most optimal case, a single ZnO film can simultaneously act as a contact, ARC, and emitter for a wafer-based silicon solar cell. Such a device would dramatically reduce the manufacture process and associated cost of solar cell production. This example simply illustrates the importance and great potential regarding the subject of granular ZnO thin films grown on crystalline silicon substrates.

It is known that due to the formation of nanometer ZnO grains, granular ZnO could increase the light-scattering capability (i.e., the haze) of the film and thus function as a light-trapping structure by enhancing the optical path and the photon absorption probability of the incident light, thus increasing the photocurrent of the solar cells [3]. As a result, for thin film solar cells, ZnO not only serve as a TCO, but also a light-trapping structure. In addition, the exposed planes in the crystal growth process depend on both kinetic and thermodynamic factors, leading to a variety of surface morphologies [11–12]. It was reported that three types of textured ZnO thin film, the columnar/polygonal, pyramid-like, and crater-like/pyramidal-tip textured structures, were grown as a front electrode in wafer-based silicon solar cells and had different influence on the performance of solar cells [11]. The ZnO(101) pyramidal-tip textured structure has been shown to perform the best with improved light-trapping and electrical properties [11]. It was shown that the formation of a pyramidal structure is mainly from the ZnO(101) plane in the hexagonal lattice [12]. Furthermore, hexagonal and pyramidal ZnO composed of the (101) and (001) planes has been synthesized in ionic liquids or obtained on Si(111) substrates by RF magnetron sputtering [13–14]. Nevertheless, the growth of well-faceted pyramidal-like ZnO on silicon substrates is still an interesting and technically challenging subject and the characterization of the physical properties of these structures has yet to be fully investigated.

In this study, textured and well-faceted ZnO thin films are grown on planar Si(100), planar Si(111), and textured Si(100) substrates by LPCVD. These three samples are characterized and analyzed by X-ray diffraction (XRD), scanning electron microscopy (SEM), atomic force microscopy (AFM), and cathode luminescence (CL) measurements. The grain structure, average grain size, and associated strains are shown to agree well with the results of the SEM, AFM, and CL measurements. Moreover, theoretical explanations for these results are given and the implications for the applications of textured and well-faceted ZnO to wafer-based silicon solar cells are discussed.

## Results and Discussion

### Structural characterization

**Figure 1:**
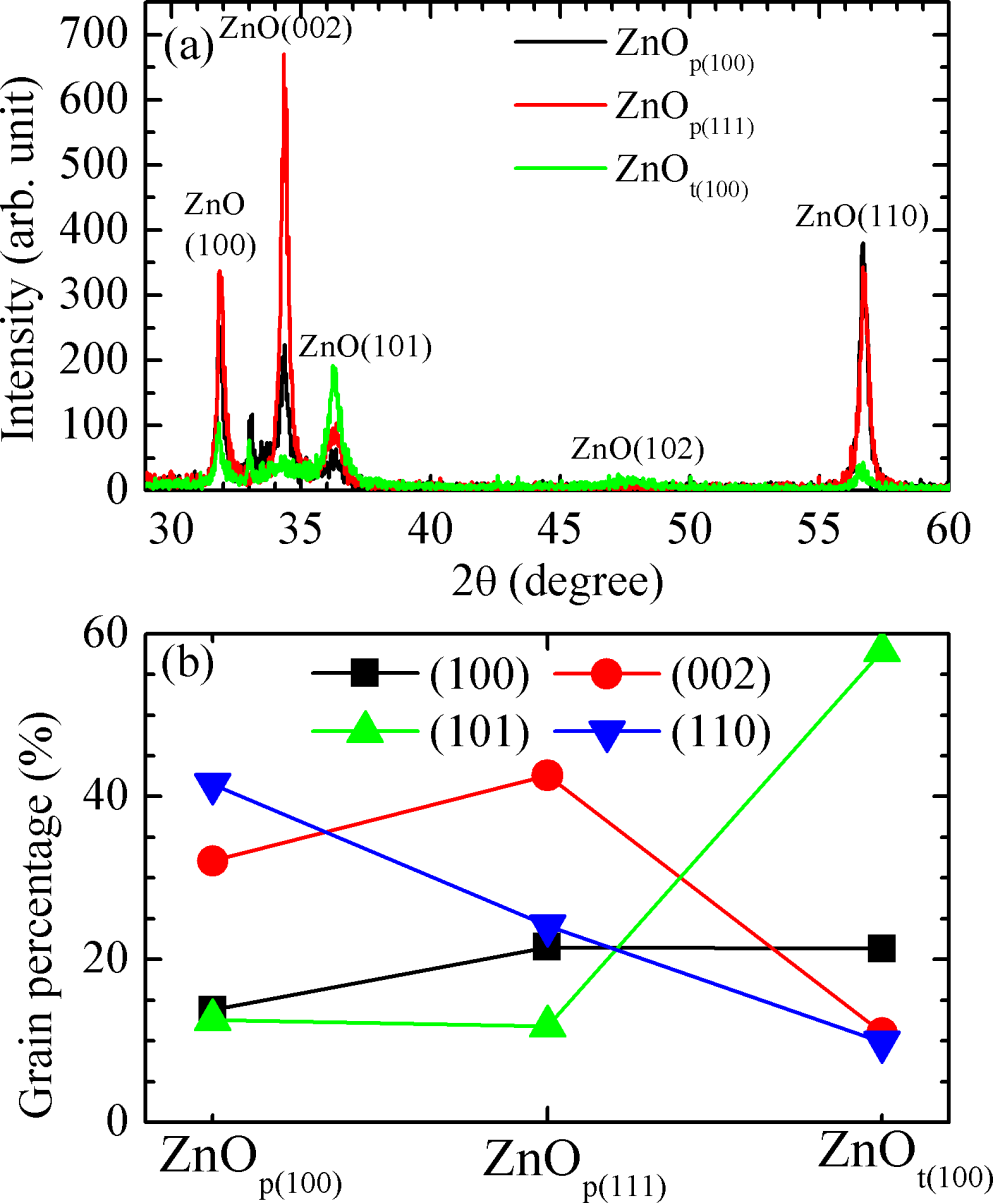
(a) XRD and (b) grain percentages obtained from integration of the intensity of the XRD peaks of the samples ZnO_p(100)_, ZnO_p(111)_, and ZnO_t(100)_.

The XRD peaks of the ZnO thin film samples grown on planar Si(100), planar Si(111), and textured Si(100) substrates, referred to hereafter as ZnO_p(100)_, ZnO_p(111)_, and ZnO_t(100)_, respectively, are shown in the Figure 1a. There are four main diffraction peaks from the crystalline ZnO: the (100), (002), (101), and (110) peaks, while the (102) diffraction peak is not very significant and will be ignored in the following discussion. If the volume percent of the different grains are assumed to be proportional to their correspondent XRD integrated intensity [15], then the grain percentages can be estimated. The percentages of different grains for the three samples are shown in Figure 1b. The dominant XRD peaks correspond to ZnO(110), ZnO(002), and ZnO(101) for the samples ZnO_p(100)_, ZnO_p(111)_, and ZnO_t(100)_, respectively.

**Figure 2:**
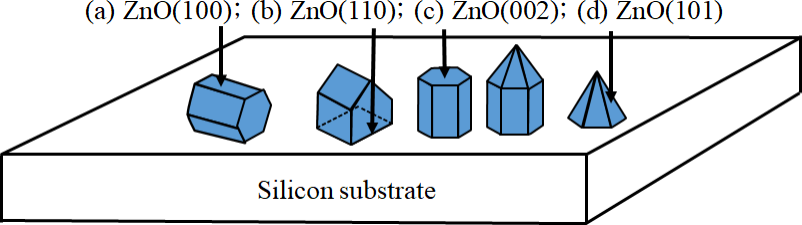
Microstructure and crystal growth orientation of ZnO grown on silicon substrates. (a) ZnO(100) on the Si(111) substrate, a polygon structure with the *c*-axis growth parallel to the substrate; (b) ZnO(110) on the Si(100) substrate, a ZnO grain with a ridge-like structure; (c) ZnO(002) on the Si(111) substrate, a ZnO grain with a pyramid-like structure (hexagonal cylinder with or without a pyramidal tip); (d) ZnO(101) on the textured Si substrate, a ZnO grain with the pyramidal tip without a hexagonal cylinder.

The exposed planes in the crystal growth process depend on both kinetic and thermodynamic factors, leading to a variety of structural morphologies [12]. The relationship between microstructure and crystal growth orientation of ZnO grown on silicon substrates is schematically illustrated in Figure 2 [12]. Here, the ZnO grains reveal a polygon structure for the *c*-axis parallel to the substrate, ridge-like structure, pyramid-like structure (hexagonal cylinder with or without the pyramidal tip), and pyramidal-tip without the hexagonal cylinder for ZnO(100), ZnO(110), ZnO(002), and ZnO(101) crystal planes, respectively.

**Figure 3:**
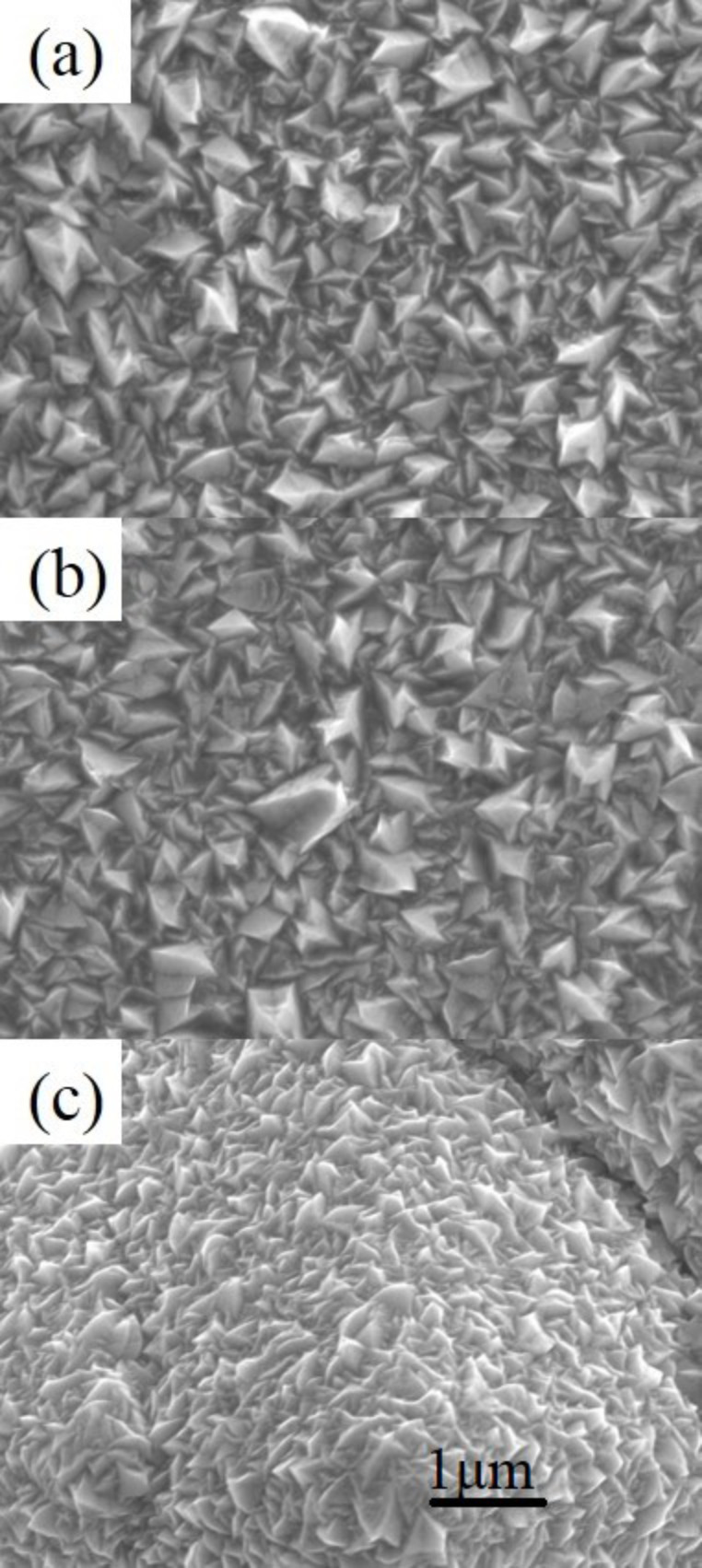
SEM images of the samples (a) ZnO_p(100)_, (b) ZnO_p(111)_, and (c) ZnO_t(100)_.

**Figure 4:**
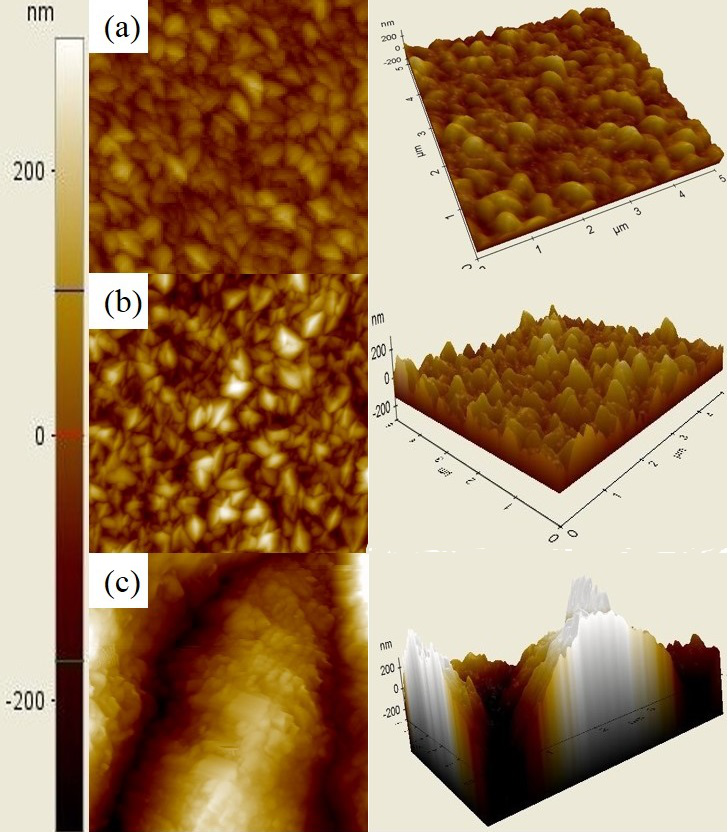
AFM images (5 × 5 μm) of the samples (a) ZnO_p(100)_ (*R*_q_: 48.488 nm), (b) ZnO_p(111)_ (*R*_q_: 48.284 nm), and (c) ZnO_t(100)_. Surface roughness of each sample, *R*_q_, is given in parentheses when available.

The surface morphologies shown in the SEM and AFM images for the three samples, shown in Figure 3 and Figure 4, respectively, can be explained by the above descriptions. The SEM images show that the ZnO_p(100)_ sample has more ridge-like structures, while the ZnO_p(111)_ sample has more pyramid-like structures. This result is consistent with the AFM images showing that the surface roughness of the ZnO_p(100)_ sample is sharper than that of the ZnO_p(111)_ sample. In addition, as shown in Table 1, the results of XRD, SEM, and AFM certainly indicate that, for the ZnO_p(100)_ sample, the dominant ZnO(110) grains show ridge-like structures on the surface; for the ZnO_p(111)_ sample, the dominant ZnO(002) grains show pyramid-like structures on the surface. For the ZnO_t(100)_ sample, the dominant ZnO(101) grains show pyramidal tips without the hexagonal cylinder on the surface.

**Table 1:** Main grain, surface morphology, surface roughness (*R*_q_) from AFM, average grain size (*D*), strain (ε), and cathode luminescence (CL) intensity of the samples ZnO_p(100)_, ZnO_p(111)_, and ZnO_t(100)_.

Sample	Main grain	Surface morphology	*R*_q_ (nm)	*D* (nm)	ε	CL intensity

ZnO_p(100)_	ZnO(110)	ridge-like structure	48.488	28.15	0.256	stronger
ZnO_p(111)_	ZnO(002)	pyramid-like structure	48.284	26.51	0.260	medium
ZnO_t(100)_	ZnO(101)	pyramid-tip structure	–	21.56	0.32	weaker

**Figure 5:**
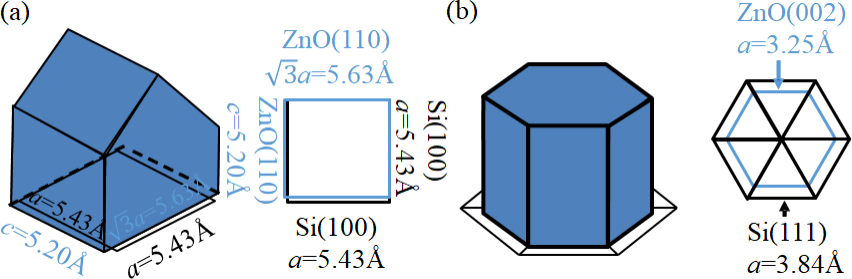
(a) The lattice mismatch between a ZnO(110) unit cell of 5.63 Å (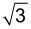
× lattice constant *a*) by 5.20 Å (lattice constant *c*) and a Si(100) cubic unit cell of 5.43 Å by 5.43 Å; (b) the lattice mismatch between a hexagonal surface of a ZnO(002) unit cell with 3.25 Å sides and a hexagonal surface of six Si(111) triangular unit cells with 3.84 Å sides.

The microstructure and crystal growth orientation of ZnO grown on different silicon substrates can be explained by the lattice mismatch between the ZnO and silicon unit cells [16]. As schematically illustrated in Figure 5a, the surface of a Si(100) cubic unit cell is a square of 5.43 Å by 5.43Å which closely matches the rectangular surface of a ZnO(110) unit cell of 5.63 Å (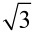
× lattice constant *a*) by 5.20 Å (lattice constant *c*). In Figure 5b, the surface of a Si(111) unit cell is a triangle of 3.84 Å. Six of these triangles form a hexagonal with sides of 3.84 Å, which closely matches the hexagonal surface of a ZnO(002) unit cell with 3.25 Å sides. It should be noted that the textured Si(100) substrate will in fact expose the Si(111) surface to form pyramid structures. However, the additional strain constraints from these Si surface textures will normally limit the growth of the ZnO(002) hexagonal cylinder and therefore favor the growth of the ZnO(101) pyramidal tips, with the same size of a surface unit cell as the ZnO(002), which closely match the textured silicon surface as well.

As a result, the planar Si(100) substrate will favor ZnO(110) crystallization, while the planar Si(111) substrate will favor Zn(002). The surface morphology of the ZnO film on planar Si(100) substrate will have more ridge-like structures from the ZnO(110) grains, while the ZnO film on planar Si(111) substrate will have more pyramid-like structures from the ZnO(002) grains.

### Average grain size and strain

**Figure 6:**
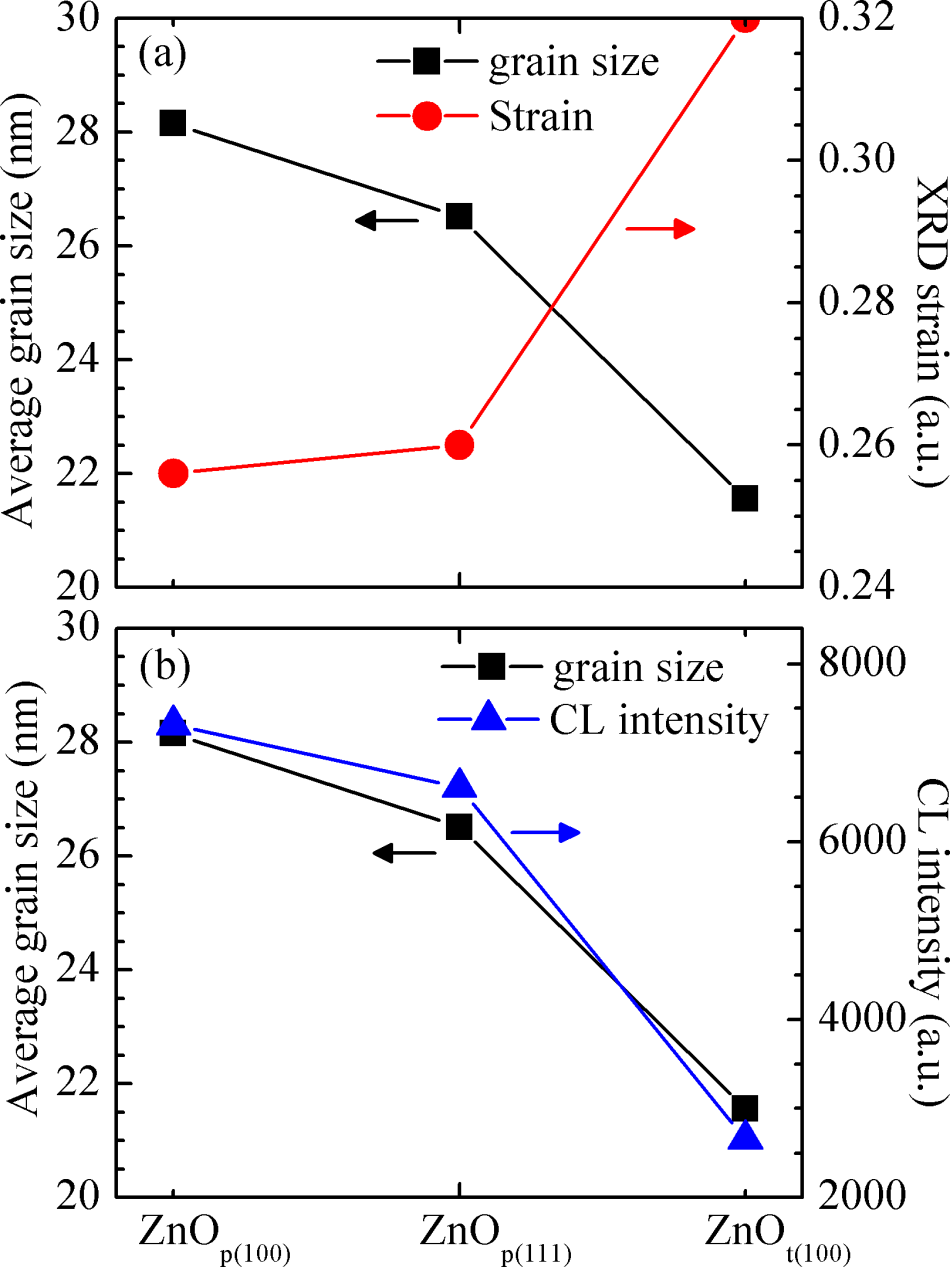
(a) Average grain size versus strain and (b) average grain size versus cathode luminescence (CL) intensity of the samples ZnO_p(100)_, ZnO_p(111)_, and ZnO_t(100)_.

In addition, the ZnO average grain size (*D*) can be calculated from the following equation [17]:

[1]
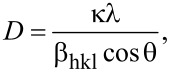


where β_hkl_, κ, λ, and θ are full width at half maximum (FWHM), shape factor (0.9), X-ray wavelength, and XRD angle, respectively. The results are shown in Figure 6a, which show that the *D* of the ZnO_p(100)_ sample is slightly larger than that of the ZnO_p(111)_ sample, while both of them are much larger than that of the ZnO_t(100)_ sample. These results can also be consistently verified by the SEM images, which show that the surface granular textures on the ZnO_p(100)_ sample are slightly larger than those on the ZnO_p(111)_ sample, while both of them are much larger than those on the ZnO_t(100)_ sample.

The XRD data can be analyzed to obtain not only the percentages of different grain crystal orientations but also the associated strain. The strain (ε) associated with the XRD peaks can be calculated by the following equation [17]:

[2]
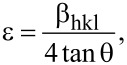


where β_hkl_ and θ are the FWHM and XRD angle, respectively. As shown in Figure 6a, the ε in the ZnO_p(100)_ sample is slightly smaller than that in the ZnO_p(111)_ sample, while both are much smaller than that in the ZnO_t(100)_ sample. The average ZnO grain sizes grown on different silicon substrates decrease with increasing strains.

### Cathode luminescence spectra

**Figure 7:**
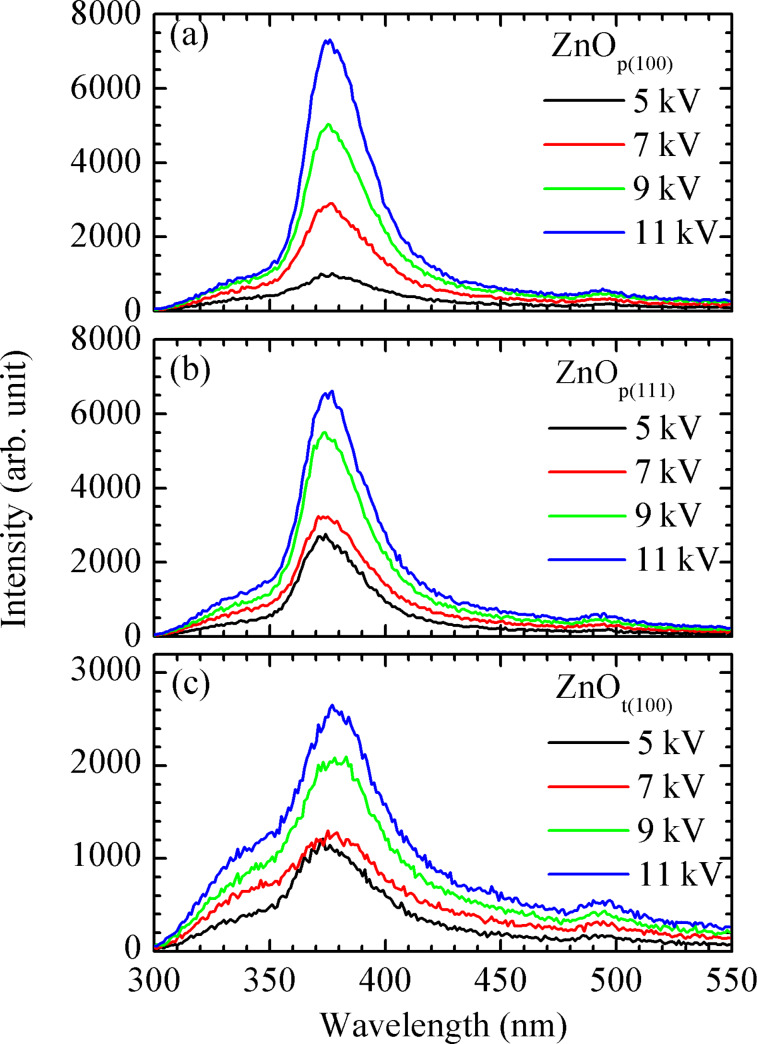
Cathode luminescence spectra of the samples (a) ZnO_p(100)_, (b) ZnO_p(111)_, and (c) ZnO_t(100)_ with the excitations of 5, 7, 9, and 11 kV electron voltages at room temperature.

In addition, the average ZnO grain size can be indirectly verified from the results of the CL measurements of the three samples at room temperature (RT), as shown in Figure 7. The ZnO films are grown to be roughly the same thickness of 1.7 μm for the three different substrates. As a result, the measured CL intensity should be proportional to the degree of crystallization of ZnO grains. An emission peak around 378 nm (3.28 eV) is related to a band-to-band transition [4–5]. The CL intensity of the sample ZnO_p(100)_ is stronger than that of the sample ZnO_p(111)_, while both of them are much stronger than that of the sample ZnO_t(100)_. Since a larger grain size corresponds to more crystalline structures and thus less defects, and this implies therefore stronger CL peak intensity, it can be expected that CL intensity is linearly proportional to the associated average grain size. As shown in Figure 6b, the average ZnO grain size estimated from XRD almost fully agrees with the CL intensity.

### Reflectance spectra

**Figure 8:**
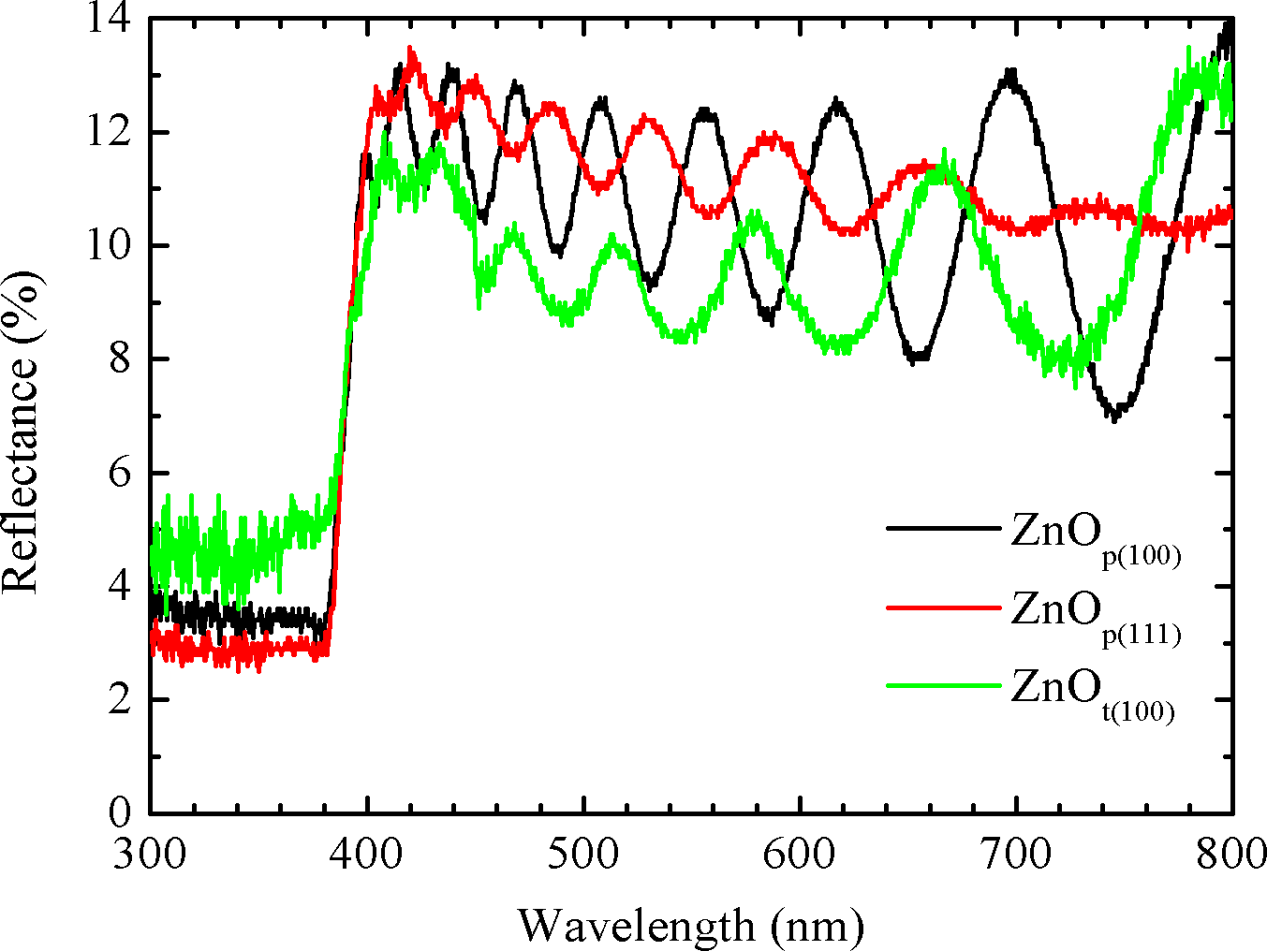
Reflectance spectra of the samples (a) ZnO_p(100)_, (b) ZnO_p(111)_, and (c) ZnO_t(100)_ at room temperature.

The reflectance spectra of the three samples were measured, as shown in Figure 8. It shows that the difference in the reflectance spectra between the ZnO_p(100)_ and ZnO_p(111)_ samples is insignificant. The ZnO_t(100)_ sample has smaller reflectance due to the additional texture provided by the micrometer-sized pyramid structure of the texture Si(100) substrate. Therefore, it could be concluded that the antireflection function provided by theses samples mostly results from the nanometer-sized texture of the ZnO films while the micrometer-sized texture of the Si substrate has a limited contribution.

## Discussion

The main grain orientation, surface morphology, AFM surface roughness (*R*_q_) from AFM, average grain size (*D*), strain (ε), and CL intensity of samples ZnO_p(100)_, ZnO_p(111)_, and ZnO_t(100)_ are shown in Table 1. The results clearly demonstrated that these results strongly agree the measurements from the SEM, AFM, and CL as well.

The results of this work show that the ZnO grown on the three different Si substrates all have stable granular structures with average grain size of 10–50 nm. Of course, in order to apply the ZnO thin films for applications in silicon solar cells, the defects resulting from the ZnO grains must be as minimal as possible. These results certainly imply that in order to minimize the defects of ZnO grains, the ZnO grain size should be as high as possible. It is worth noting that the ZnO samples grown on silicon substrates presented in this work have the potential to be a cost-effective alternative material for the substitution of the indium tin oxide (ITO) thin layer in heterojunction with intrinsic thin layer (HIT) solar cells [18]. Nevertheless, the optimization of the LPCVD growth conditions and parameters to increase the ZnO grain size and minimize their associated defects is an interesting issue for further studies and investigations.

## Conclusion

In this work, textured and well-faceted ZnO samples are grown on planar Si(100), planar Si(111), and textured Si(100) substrates by the LPCVD method. Due to the constraints of the lattice mismatch between the ZnO and Si crystal structures, the ZnO_p(100)_, ZnO_p(111)_, and ZnO_t(100)_ samples favor the growth of ZnO(110) ridge-like, ZnO(002) pyramid-like, and ZnO(101) pyramidal-tip structures, respectively. The average ZnO grain size on the planar Si(100) substrate is slightly larger than that on the planar Si(111) substrate, while both of them are much larger than that on the textured Si(100) substrate. It has been shown that the average grain sizes of ZnO grown on the different silicon substrates decrease due to the increases in the corresponding strain. Although this particular research result is not new, this work indeed provides useful information for optimized growth of textured and well-faceted ZnO grown on wafer-based silicon solar cells. These results can be utilized for efficiency enhancement by optimizing device structures, such as HIT solar cells, where the ZnO could be a cost-effective alternative material for ITO.

## Experimental

### Synthesis of ZnO on silicon substrates by LPCVD

Three different Si substrates, planar (100), planar (111), and textured (100), were prepared. These Si substrates were dipped in 5% HF solution to remove the native oxide layer and then rinsed in deionized water in 3 min. The ZnO films with the thickness of 1.7 μm were deposited on planar (100), planar (111), and textured (100) Si substrates (simply denoted as samples ZnO_p(100)_, ZnO_p(111)_, and ZnO_t(100)_, respectively) at a temperature of 170 °C by a LPCVD process. Diethylzinc (DEZ) and water (H_2_O) vapors carried by argon gas were used as precursors, and their flow rates were set to 500 and 550 sccm, respectively. The textured Si(100) substrate was prepared from a monocrystalline Si(100) wafer anisotropically etched in NaOH/IPA solution at 85 °C to form pyramidal structures at the wafer surface. The surface roughness of these pyramidal structures was measured as 0.226 µm using AFM. Therefore, the average size of the pyramidal structures was taken to be approximately this value. The pyramidal structures will expose their (111) facets to the wafer surface. However, their physical properties for the LPCVD growth the ZnO films will be different from the (111) facets of the planar Si(111) substrate due to their different surface morphologies, especially, the additional strain introduced for ZnO films at the peaks and valleys of the pyramidal structures.

### Characterization

The structures of the samples ZnO_p(100)_, ZnO_p(111)_, and ZnO_t(100)_ were investigated with a high-resolution XRD (Bede D1). The surface morphology was revealed by atomic force microscopy (Park Systems, XE-70) operating in non-contact mode using a silicon tip of curvature less than 10 nm. Scanning electron microscope and cathode luminescence results were acquired by the use of a Gatan monoCL3 spectrometer in a JEOL JSM 7000F SEM system.
